# Degradation Kinetics of Benzyl Nicotinate in Aqueous Solution

**DOI:** 10.4103/0250-474X.62238

**Published:** 2010

**Authors:** C. J. Mbah

**Affiliations:** Department of Pharmaceutical and Medicinal Chemistry, Faculty of Pharmaceutical Sciences, University of Nigeria, Nsukka, Enugu State, Nigeria

**Keywords:** Degradation kinetics, benzyl nicotinate

## Abstract

The degradation of benzyl nicotinate in aqueous solution over a pH range of 2.0-10.0 at 50±0.2° was studied. The degradation was determined by high performance liquid chromatography. The degradation was observed to follow apparent first-order rate kinetics and the rate constant for the decomposition at 25° was estimated by extrapolation. The reaction was shown to be hydroxide ion catalyzed and the Arrhenius plots showed the temperature dependence of benzyl nicotinate degradation. A significant increase in the stability of benzyl nicotinate was observed when glycerol or polyethylene glycol 400 was incorporated into the aqueous solution.

Benzyl nicotinate, 3-pyridine carboxylic acid phenylmethyl ester is a rubefacient. It acts by counter irritation to relief pain muscles, joints and other non-articular musculoskeletal conditions. Benzyl nicotinate has been shown to improve skin oxygenation[1], induce dermatitis artefacta[2], cause hyperemia[3], stimulate percutaneous absorption[4]. A dermal vasodilating effect of benzyl nicotinate has also been reported[5,6]. Recently, it has been incorporated into some cosmetic products as a lip plumber due to its ability to increase blood circulation in the lip area. As the efficacy of a cosmeceutical or pharmaceutical preparation depends on the stability of its active ingredients, the objectives of this study were to (i) investigate the degradation rate of benzyl nicotinate in aqueous solution with the view of understanding its stability in a cosmetic product. (ii) Investigate the influence of non-aqueous solvents very often used as vehicles or co-vehicles in cosmetic products manufacture on the rate of degradation of benzyl nicotinate. A review of the literature showed little or no report on the hydrolysis of benzyl nicotinate in aqueous solution. In this paper, we report on the degradation kinetics of benzyl nicotinate in various buffer solutions and cosolvent systems by high performance liquid chromatography.

## MATERIALS AND METHODS

Benzyl nicotinate was received from EMD Chemicals Inc., New Jersey, USA. Buffer substances, organic solvents and all other chemicals used were of analytical grade.

### Chromatography:

All separations were carried out with Hitachi LC-6200 pump, AS 2000 autosampler, Kratos spectroflow 783 detector (Spectra Physics, USA) and Zorbax analytical column SB-CN 150 × 4.6 mm, 3.5 μm (Agilent Technologies, USA). pH measurement was performed with ThermoOrion pH meter, model 330 with combination glass electrode. The mobile phase consisted of methanol-water-acetic acid (50:50:1) and was degassed by vaccum pump filtration. The flow rate was 1 ml/min at room temperature. The injection volume was 10 μl and the effluent was detected at 254 nm.

### Buffer and Standard solutions:

The buffer solutions were KCl-HCl, pH 2-3; NaOH-KH_2_ PO_4_, pH 7.4 and H_3_ BO_3_ -NaOH-KCl, pH, 8-10. Constant ionic strength (μ) of 0.4 mol/l was maintained for each buffer by adding calculated amount of potassium chloride. The solutions were freshly prepared and the pH values were determined with pH meter (ThermoOrion, USA) equipped with a combination glass electrode.

Stock solution of benzyl nicotinate (800 μg/ml) was prepared in methanol. Aliquots of the standard stock solution were pipetted in to a 10 ml volumetric flask and diluted to volume with methanol to give final concentration of 80-400 μg/ml benzyl nicotinate.

### Kinetic measurement[7]:

The rate studies were performed in buffer solutions at 50±0.2°. The total buffer concentration was 0.1 mol/l. Stock solution of benzyl nicotinate was diluted with buffer solution to give a concentration of 400 μg/ml and the solutions were kept in a water bath. At appropriate intervals, aliquots were withdrawn and injected into the chromatograph. The rate constants were determined from the slopes of linear plots of logarithm of percent benzyl nicotinate remaining versus time. The effect of temperature on the hydrolytic reaction of benzyl nicotinate was determined at temperatures ranging from 50 to 80°. The solvent effect on the hydrolysis of the drug was also determined in different solvent:water ratios (5:95; 10:90; 15:85 and 20:80) of glycerol:water or polyethylene glycol 400:water systems. All solutions of the cosolvent systems were buffered at pH 10.0 with borate buffer.

## RESULTS AND DISCUSSION

**Fig. 1:**
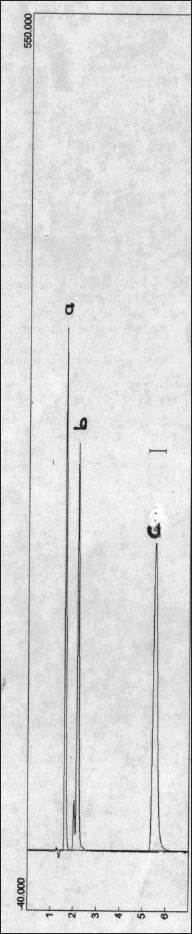
Chromatogram of benzyl nicotinate and its degraded products. a and b = degraded products; c = benzyl nicotinate.

**Fig. 2:**
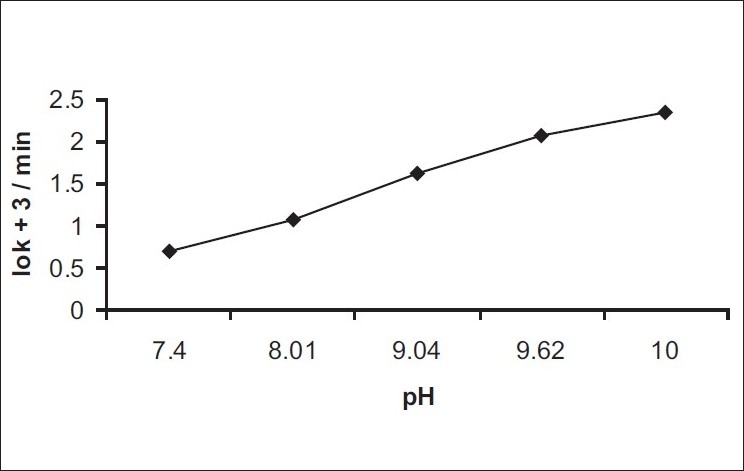
Plot of logarithm of observed first-order rate constant versus pH. The curve (■-----■) represents pH 7.4-10.0

The calibration graph of benzyl nicotinate was linear in the concentration range of 80-400 μg/ml. The correlation coefficient obtained from the regression equation describing the peak area versus concentration relationship is 0.9998. The kinetics of degradation of benzyl nicotinate was studied at 50±0.2° over the pH range of 2.0-10.0. At pH 2.0-3.0, no degradation of benzyl nicotinate was observed, however at pH values of 7.4-10.0 where degradation occurred, the degradation was observed to be of pseudo first-order rate kinetics. The results are presented in (Table 1). The degradation was followed until less than 10% of benzyl nicotinate height remained. A typical chromatogram obtained from the hydrolytic degradation of benzyl nicotinate is shown in (fig. 1). The pH-rate profile for the degradation of benzyl nicotinate at 50±0.2° is shown in (fig. 2). The plot was constructed from the logarithm of the observed pseudo first-order rate constants and the corresponding pH values. The graph obtained indicated hydroxide ion catalyzed degradation. In the studied pH range, a general hypothetical rate equation for benzyl nicotinate decomposition as a function of pH can be written as, k_obs_ = k_o_+k_OH_ [OH^−^], where k_obs_ is the overall observed rate constant, k_o_ is the water catalysis rate constant, k_OH_ is the hydroxide ion catalysis rate constant. The apparent first-order rate constant for water catalyzed and the second-order rate constant for hydroxide ion catalyzed decomposition were determined to be 0.0149/min and 2141/mol/min, respectively.

**TABLE 1:** FIRST-ORDER RATE CONSTANT OF BENZYL NICOTINATE IN AQUEOUS SOLUTION

pH	Buffer	k_obs_ (min^−1^)^x^	t_1/2 (min)_
2.02	Hydrochloric acid	-	-
3.04	Hydrochloric acid	-	-
7.40	Phosphate	0.0051±4.32	135.9
8.01	Borate	0.0116±1.07	59.7
9.04	Borate	0.0432±0.51	16.0
9.62	Borate	0.1159±0.22	6.0
10.00	Borate	0.2221±0.14	3.1

Temperature: 50 ± 0.2°; Ionic strength (μ): 0.4 mol/l

x Mean ± RSD (%); n = 3

The catalytic effect of buffer species used in the kinetic investigation was determined at constant pH (9.04), ionic strength (μ=0.4) and temperature (50±0.2°) and varying only the buffer concentration. The results (Table 2) showed that the rate constant was unaffected by buffer concentration, indicating lack of general base catalysis. The effect of ionic strength on the degradation of benzyl nicotinate was also studied by keeping the buffer concentration (0.1 mol/l), pH (7.40) and temperature (50±0.2°) constant and varying only the ionic strength by the addition of different amounts of potassium chloride. The results (Table 2) indicate no kinetic salt effect for the degradation of benzyl nicotinate.

**TABLE 2:** EFFECT OF BUFFER CONCENTRATION AND IONIC STRENGTH ON THE FIRST-ORDER RATE CONSTANT OF BENZYL NICOTINATE

Buffer concentration		Ionic strength	
	
(mol/l)^y^	k_obs_ (min^−1^)^x^	KCl (mol/l)^z^	k_obs_ (min^−1^)^x^
0.05	0.0447±6.21	0.200	0.0054±3.17
0.10	0.0421±6.87	0.400	0.0048±4.36
0.15	0.0438±5.94	0.600	0.0056±2.94
0.200	0.0453±4.52	0.800	0.0052±4.02

y 50±0.2°; pH 9.04; H_3_BO_3_-NaOH-KCl

z 50±0.2°; pH 7.40; NaOH-KH_2_PO_4_;

x Mean±RSD (%); n=3

**Fig. 3:**
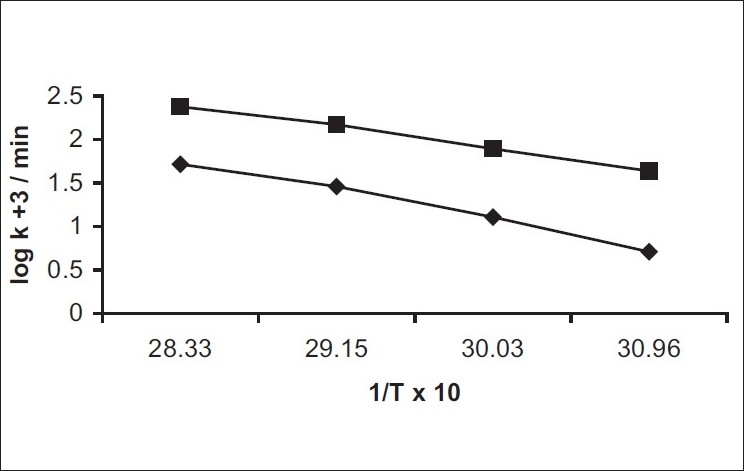
(log k_obs_+3 versus 1/T×10^4^); upper curve (■-----■) represents pH 9.04; lower curve (♦-----(♦) represents pH 7.40

The influence of temperature on the degradation of benzyl nicotinate was studied by measuring the apparent first-order rate constant at pH 7.40 and 9.04, respectively, constant ionic strength (μ = 0.4) and temperature ranging from 50 to 80°. The results are given in Table 3. A plot of the logarithm of the observed first-order rate constant against the reciprocal of the absolute temperature is shown in fig. 3. The Arrhenius plots were linear (r>0.9892) indicating that the degradation mechanism was not with a change in temperature within the temperature range studied. This showed single degradation mechanism which justified the extrapolation of the results to obtain rate constants at 25°. The activation energy for hydrolytic reaction was evaluated using the Arrhenius equation. The activation energies are 70.7 kJ/mol (pH 7.40) and 55.0 kJ/mol (pH 9.04), respectively. The estimated rate constants at 25° were 0.0007/min (pH 7.40) and 0.0079/min (pH 9.04), respectively.

**TABLE 3:** EFFECT OF TEMPERATURE ON THE FIRST-ORDER RATE CONSTANT OF BENZYL NICOTINATE

pH	Temperature (°)	k_obs_ (min^−1^)^x^	t_1/2_ (min)
7.40	25	0.0007	990.0
	50	0.0051±4.32	135.9
	60	0.0128±1.93	54.1
	70	0.0289±1.24	24.0
	80	0.0520±0.57	13.3
9.04	25	0.0079	87.7
	50	0.0432±0.59	16.0
	60	0.0782±0.31	8.9
	70	0.1482±0.21	4.7
	80	0.2371±0.12	2.9

x Mean±RSD (%); n=3

Using the rate constants at 25°, the frequency factors were evaluated to be 1.62×10^9^ /min (pH 7.40) and 3.41×10^7^ /min (pH 9.04), respectively. The high values of the frequency factors indicate a large proportion of collisions between benzyl nicotinate molecules and hydroxide ions during the hydrolytic reactions. The values also showed that most effective collisions occurred at pH 7.40 than pH 9.04. The half-lives of degradation at 25° were found to be 990 min (pH 7.40) and 88 min (pH 9.04), respectively.

**Fig. 4:**
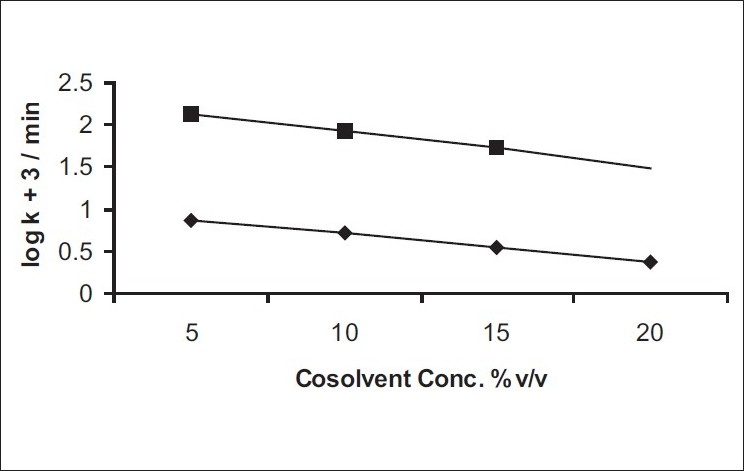
Plot of logarithm of observed first-order rate constant versus cosolvent concentration. The upper curve (■-----■) represents glycerol effect; lower curve (♦----♦) represents polyethylene glycol 400 effect

The results of the effect of non-aqueous solvents on the degradation of benzyl nicotinate using different ratios of glycerol:water or polyethylene glycol 400:water systems are given in Table 4. The degradation of benzyl nicotinate in these cosolvent systems followed first-order rate kinetics. The rate constant decreased as the content of glycerol or polyethylene glycol 400 in the solution was increased. It was also observed that polyethylene glycol 400 showed more decreasing effect on the rate constant of benzyl nicotinate than glycerol. The stabilization effect of the cosolvent on benzyl nicotinate degradation may perhaps be due to decrease in dielectric constant and viscosity increase of the solutions. A plot of logarithm of the rate constant versus cosolvent concentration (fig. 4) showed a linear relationship (r> 0.998).

**TABLE 4:** EFFECT OF NONAQUEOUS SOLVENTS ON THE FIRST-ORDER RATE CONSTANT OF BENZYL NICOTINATE

Concentration of cosolvent (% w/v)	Glycerol		Polyethylene glycol 400	
		
	k_obs_ (min^−1^)^x^	t_1/2_ (min)	k_obs_ (min^−1^)^x^	t_1/2_ (min)
5	0.1335±0.30	5.2	0.0074±5.26	93.6
10	0.0853±0.35	8.1	0.0052±5.66	133.3
15	0.0529±0.57	13.1	0.0035±11.02	198.0
20	0.0529±0.57	13.1	0.0023±12.8	301.3

Temperature: 50±0.2°

x Mean±RSD (%); n=3

In this investigation, no attempt was made to characterize the degradation products; however, a plausible reaction mechanism of the hydrolysis is a nucleophilic attack by hydroxide ion on the electron deficient carbon of the carbonyl group followed by the cleavage of the benzyloxy group to give benzyl alcohol and nicotinic acid.

In conclusion, the breakdown of benzyl nicotinate was found to be first-order rate kinetics and the hydrolytic reaction was hydroxide ion catalyzed. Buffer concentration or ionic strength had no effect on the rate constant. Half-lives were evaluated from the constants obtained using Arrhenius equation. Polyethylene glycol 400 exhibited greater stabilizing properties on the rate constant of benzyl nicotinate than glycerol. Finally, the study showed that the incorporation of glycerol or polyethylene glycol 400 into cosmetic product containing benzyl nicotinate would enhance the stability of the drug even under alkaline conditions.
